# Prospective Association Between Illness Uncertainty and Self-Management Self-Efficacy in Patients With Early-Stage Chronic Liver Disease Over a Six-Month Follow-Up

**DOI:** 10.7759/cureus.92787

**Published:** 2025-09-20

**Authors:** Habeeb Adnan Farooqui, Muhammad Hassaan Zafar, Ayesha Haider, Abdul Samad, Saif Ur R Qureshi, Muhammad Adnan Shahid, Aman Shazib, Addan Farooq, Nadia Naz, Zain Bin Saeed, Muhammad Maaz

**Affiliations:** 1 Internal Medicine, Shadan Institute of Medical Sciences, Hyderabad, IND; 2 Paediatrics and Neonatology, Medicare Hospital, Multan, PAK; 3 Emergency Department, Mukhtar A Sheikh Hospital, Multan, PAK; 4 Medicine, King Edward Medical University, Lahore, PAK; 5 Internal Medicine, Liaquat University of Medical and Health Sciences, Hyderabad, PAK; 6 Medicine, Liaquat University of Medical and Health Sciences, Hyderabad, PAK; 7 General Medicine, Blackpool Teaching Hospitals, Blackpool, GBR; 8 Internal Medicine, Allama Iqbal Medical College, Lahore, PAK; 9 Medicine, Bahria Medical and Dental College, Karachi, PAK; 10 Medicine, Bolan Medical College, Quetta, PAK; 11 Medicine/Internal Medicine, Allama Iqbal Medical College, Lahore, PAK; 12 Medicine, Watim Medical College Rawat, Islamabad, PAK

**Keywords:** chronic disease management, chronic liver disease, illness uncertainty, mishel uncertainty in illness scale, self-efficacy, self-management behaviors

## Abstract

Background: Chronic liver disease (CLD) is a long-term, frequently progressive disease with few indicators of the disease during the initial phases. Many patients face uncertainty regarding their illness, and it might have adverse impacts on their confidence in dealing with being ill. This study aimed to examine the prospective association between illness uncertainty and self-management self-efficacy over six months in patients with early-stage CLD. We hypothesized that higher baseline illness uncertainty would predict lower self-efficacy over time, whereas higher baseline self-efficacy would predict better future self-management confidence.

Methods: The study involved a prospective observational study of 384 patients with early-stage CLD who were recruited across outpatient hepatology clinics in Islamabad, Pakistan. Illness uncertainty was assessed at baseline using the Mishel Uncertainty in Illness Scale - Community Form (MUIS-C), and self-management self-efficacy was evaluated at baseline, three months, and six months using the Self-Efficacy for Managing Chronic Disease 6-Item Scale (SEMCD-6), which measures patients' confidence in performing self-management tasks rather than actual behaviors. Descriptive statistics, Pearson correlations, independent t-tests, repeated measures ANOVA, and multiple linear regression were used to analyze the data, with mean differences between groups indicating statistical significance at p < 0.05. Data collection took place from July 2024 to December 2024, spanning a six-month period.

Results: Illness uncertainty was significantly related to lower self-efficacy in three time points (T1, T2, and T3; p < 0.001). The results showed that female participants had greater illness uncertainty and lower self-efficacy compared to male participants (p < 0.001). There was a small but significant improvement in self-efficacy scores after six months (p < 0.001). Baseline illness uncertainty was found to be an important negative predictor of self-efficacy at six months (B = -0.06, p = 0.001), while baseline self-efficacy emerged as the most potent positive predictor (B = 0.55, p < 0.001) according to regression analysis. The kind of liver disease had a small, significant impact, and comorbidities were not influential predictors.

Conclusion: Illness uncertainty negatively influences self-management self-efficacy in patients with early-stage CLD, although self-efficacy slightly improves over time. Reducing illness uncertainty early can play a crucial role in enhancing self-efficacy and promoting positive long-term health outcomes. Educational and psychological support strategies, as part of preventive efforts, must be highlighted when planning a hepatology patient intervention.

## Introduction

Chronic liver disease (CLD) is a rising global health issue. It contributes to mortality, morbidity, and the costs incurred by people and organizations, with hospitalizations having increased by 92% over the past decade [1,2]. Smoking, alcohol intake, lack of activity, and central obesity are some of the high-risk lifestyle determinants for the development of CLD, cardiometabolic disease, and death. The risk of CLD increased by 30% with every additional risk factor and further deteriorated outcomes at every level of disease [3].

High illness uncertainty is closely associated with low quality of life among patients with nonalcoholic fatty liver disease (NAFLD) [4]. The uncertainty of illness, defined by the lack of control and doubt, leads to maladaptive coping, greater psychological distress, and loss of quality of life. This pervasive uncertainty impacts both patients and caregivers in advanced liver disease, exacerbating distress and complicating care planning, highlighting targeted supportive care interventions [5,6].

In end-stage liver disease (ESLD), increased illness uncertainty and disease severity correlate with increased completion of advance directives (ADs), whereas avoidant coping was negatively correlated with AD completion [7]. Depression, disease severity, and self-efficacy in liver cirrhosis patients are essential factors in defining self-management behaviors. Increasing self-efficacy and reducing psychological distress may lead to improved adherence to self-care strategies and better overall coping with the disease [8].

NAFLD patients display poor self-management practices, such as regarding disease knowledge, with variables such as gender, educational level, BMI, and sleep quality causing variations [9]. Following liver transplant, young people exhibit diminished independence in self-management, yet improved adherence to medications when compared with peers with autoimmune or other CLDs [10]. In a cohort study of 159 patients with alcohol-related liver disease, patients' beliefs about symptoms, understanding of their illness, and concerns were significantly associated with self-efficacy (confidence in managing their condition), quality of life, and psychological well-being over the course of their outpatient follow-up visits [11].

Rationale

Patients at an early stage of CLD have trouble managing their disease since the disease can be silent or unpredictable during the initial stages. Numerous patients experience uncertainty about the meaning of their condition, or about the severity of their illness, or what they need to do to remain healthy. Such a feeling of doubt can influence the personal choices they make every day, making them less willing to adhere to identified health community practices, such as eating healthier, exercising, taking medication on time, or visiting a doctor periodically.

Consequently, there is little evidence on the longitudinal impact of uncertainty about illness on self-management behaviors in CLD. The majority of individuals only turn to help once complications aggravate, a time that is probably too late to avoid complications. The research has the potential to identify early indicators of unfavorable disease management, as studying how illness uncertainty affects self-care behaviors relies on months-long assessment periods. This knowledge can help healthcare providers provide more effective support and guidance, enabling patients to feel motivated and take steps to maintain their health.

Primary objective

The primary objective of the study was to investigate the prospective relationship between illness uncertainty and self-management self-efficacy of patients with early-stage CLD over a six-month follow-up period.

Secondary objectives

Secondary objectives were to assess whether self-management self-efficacy changes over time, examine which aspects of self-efficacy are most affected by baseline illness uncertainty, and determine the predictive value of baseline illness uncertainty on subsequent self-efficacy.

## Materials and methods

Research design and methods

In this study, a prospective observational research design was employed to investigate the correlation between illness uncertainty and self-management in patients with early-stage CLD over a six-month follow-up period. Two outpatient hepatology hospitals in Islamabad, Pakistan, namely Shifa International Hospital and Pakistan Institute of Medical Sciences (PIMS), were selected to recruit a sample size of 384. This employment method ensured representation from a broad range of socioeconomic and educational backgrounds, helping to produce a more inclusive body of patients.

Structured questionnaires, consisting of a demographic section, the Standard Illness Uncertainty Scale, and a validated self-management behavior questionnaire specific to CLD, were used to collect data. The Illness Certainty Questionnaire was administered once at baseline. The self-management questionnaire was administered at three time points: at baseline, three months, and six months. This design enabled the researchers to detect any trends, variations, and patterns of self-management behavior over time, in contrast to the baseline levels of illness uncertainty. While this approach enabled the study of how initial psychological factors were associated with subsequent self-management behavior, changes in illness uncertainty over time could not be assessed, limiting causal inferences about its effects on behavior.

Sampling size and sampling technique

It was estimated that the study had an infinite population because there is no definite information on the total number of individuals with early-stage CLD who have varied degrees of illness uncertainty or variances in self-management thoughts. The required sample size was calculated as follows:

\[n = \frac{Z^2 \cdot p (1 - p)}{d^2}\]

*Z* in this equation is the standard score at the desired level of confidence, *p* is the assumed proportion, and *d* is the margin of error. The margin of error used was 0.05 (*Z* = 1.96), corresponding to a 95% confidence level. To give the highest necessary sample size, *p* was set as 0.50 [12].

According to these parameters, the sample size was calculated to be 384 individuals. Participants in the outpatient hepatology hospitals were recruited using a convenience sampling method. This non-probability sampling method enabled us to select individuals who were eligible and easy to reach, and who were willing to participate in the study. The sample size was determined using the standard formula for estimating proportions, ensuring adequate precision at a 95% confidence level with a 5% margin of error.

Inclusion criteria

The study participants were adult individuals aged 18 years and above who had been clinically diagnosed with the early stage of CLDs, including NAFLD, chronic hepatitis, or mild fibrosis. Patients on regular outpatient follow-ups at the early and non-cirrhotic stages of the liver disease were taken as eligible ones only. Additionally, participants were required to be able to read and answer questionnaires independently or with minimal assistance, and informed consent was obtained before their participation.

Exclusion criteria

The study excluded patients diagnosed with advanced liver disease, such as cirrhosis or hepatocellular carcinoma, or a history of liver transplant. Patients with serious comorbidities that may have been able to influence self-management independently, including severe psychiatric disease or cognitive impairment, were also excluded. Moreover, patients who could not make follow-up checks because of mobility issues, language differences, or a lack of willingness to undergo multiple follow-ups were not included in the final sample.

Data collection tools

A structured questionnaire was employed in this study, consisting of three sections: demographic details, measurement of illness uncertainty, and measurement of self-management behaviors regarding early-stage CLD. Validated, standardized scales were used as instruments, together with demographic questions developed by the researchers to ensure a rich measurement of the study variables. No standardized tool was translated; instead, it was employed in its original English language form.

Demographic information

The initial part of the questionnaire gathered extensive demographic and clinical data to examine possible relationships between the characteristics of the participants and the differences in self-management practices. The following data were collected: age, gender, marital status, education level, and employment status. Besides these, clinical information included timelines since the diagnosis of liver disease, the type of liver disease as indicated by the physician, the current status of treatment, and the occurrence of comorbid diseases. This section enables the provision of a thorough descriptive assessment of the participants, thereby facilitating an investigation of subgroup variations within the study population.

Mishel Uncertainty in Illness Scale - Community Form (MUIS-C)

The Mishel Uncertainty in Illness Scale (MUIS) was developed by Merle H. Mishel (1981) to measure uncertainty in illness. In this study, the community form (MUIS-C) was used, comprising a total of 23 items. Each rated on a five-point Likert scale, ranging from 1 (strongly disagree) to 5 (strongly agree). A total score is determined by adding all item responses, with higher scores indicating a greater perceived uncertainty about illness. The scale consists of both a set of positive and negative items; negative statements are reverse-scored, and then the total score is computed. Negative items were reverse-coded before summing to produce a total score. Higher scores indicate greater perceived illness uncertainty. When ≤1 item was missing, the mean of completed items was used for imputation. The MUIS-C has excellent internal reliability, as indicated by a Cronbach's alpha of approximately 0.85, which demonstrates good reliability. In this research study, MUIS-C was conducted in its original English form without translation [13].

Self-Efficacy for Managing Chronic Disease 6-Item Scale (SEMCD-6)

Self-efficacy in managing chronic disease was assessed using the SEMCD-6, developed by Kate Lorig and colleagues at Stanford University in 2001. The instrument evaluates how patients feel about their capabilities to cope with different situations of living with a chronic condition. The SEMCD-6 is composed of six items, and the responses are measured on a 10-point Likert scale where 1 is equivalent to not at all confident and 10 is equivalent to totally confident. To compute an overall score, the average of the scores of the six items is calculated, and the higher the score, the greater the self-efficacy in managing chronic disease. Overall self-efficacy scores were computed as the average of the six items. Missing responses were handled by averaging available items if ≤1 item was missing; otherwise, the participant’s data were excluded from analysis for that time point. This scale identifies the significant areas in self-management, such as symptom control, role functioning, and emotional well-being. The SEMCD-6 has demonstrated excellent internal consistency, with a Cronbach's alpha commonly reported to be 0.91, indicating high reliability of the instrument across various individuals with chronic illnesses. There was no translation of the SEMCD-6 in this research; however, the instrument was administered in its original English format [14]. This was achieved by collecting data on three occasions, i.e., baseline, three months, and six months, to evaluate the variation in self-management confidence over time.

Procedure

Participants in the study were identified through direct invitation when they were present as regular outpatients in the selected hepatology clinics in Islamabad. The participants became part of the study after providing informed written consent, following a thorough briefing on the study's purpose and procedure. The data collection took place from July 2024 to December 2024, spanning six months. Participants were asked to complete a standardized self-administered questionnaire, which involved demographic information, the MUIS-C, and the SEMCD-6 at the baseline visit. The follow-up evaluations were conducted at three and six months, during which only the SEMCD-6 was readministered to monitor changes in self-management patterns. The respondents were given the freedom to complete the questionnaires either independently or with the assistance of a trained research assistant, based on their understanding and comfort level. To ensure that the responses remained confidential, their identities were kept anonymous, and no personal details were linked to the information. This method facilitated the ethical collection of data and enabled the inclusion of a diverse range of participants from various socioeconomic and educational backgrounds.

Statistical analysis

The data analysis was performed using IBM SPSS Statistics version 26 (IBM Corp., Armonk, NY). Descriptive statistics were used for participant characteristics. The Kolmogorov-Smirnov and Shapiro-Wilk tests assessed normality. Pearson correlations assessed the association between illness uncertainty and self-efficacy at three time points (T1, T2, and T3). Independent samples t-tests examined gender differences, expressed as Cohen's d. Repeated measures ANOVA (with Greenhouse-Geisser correction for violations of sphericity) analyzed changes in self-efficacy over time. Multiple linear regression was conducted to explore associations between baseline illness uncertainty, self-efficacy, liver disease type, comorbidities, and follow-up self-efficacy, acknowledging that causality cannot be inferred due to the observational design. Chi-square tests examined age, comorbidities, and liver disease type associations. Significance was set at p < 0.05.

The study recruited 410 participants to consider any non-response/missing data. The listwise deletion was applied to eliminate incomplete responses, and the data of 384 participants were analyzed. Each of the statistical tests was performed at a significance level of p < 0.05.

Ethical considerations

All ethical regulations relating to human research were observed in the study. Before data were collected, ethical consent was granted by the Institutional Review Board (IRB) of Watim Medical College, Islamabad (WMCR/ERB/2024/28). The approval showed that this research fulfilled the requirements of key ethical values, such as respect for people, preservation of the interests of the study members, and confidentiality of personal data. The aim of the research, procedures utilized, risks, and expected benefits were all fully explained to all the participants. All participants signed the written informed consent before enrollment in the study.

The nature of participation in the study was strictly voluntary, and participants were well informed of their ability to withdraw from the study without any repercussions. The answers remained anonymous and were not to be used in any other way than research and academics. Missing data were addressed responsibly; minor cases of random missing data were addressed using listwise deletion, and highly incomplete cases were altogether omitted to make the data reliable and accurate.

## Results

Table 1 shows the demographic and clinical characteristics of the subjects (N = 384). Most of the participants were aged between 36 and 45 years (n = 118, 31%), between 46 and 60 years (n = 118, 31%), and 60 years and above (n = 82, 21%). The participants were predominantly women (n = 214, 56.0%), followed by men (n = 170, 44.0%). In terms of marital status, there were high percentages of people who were divorced (n = 145, 38%), married (n = 109, 28%), widowed (n = 80, 21%), and single (n = 50, 13%). Regarding educational levels, the majority had higher secondary education (n = 88, 23%), followed by secondary education (n = 84, 22%), with 70 (18%) participants having a bachelor's degree and 48 (12%) having a master's level degree. In terms of employment, 126 (33%) were unemployed, 106 (28%) were self-employed, 92 (24%) were retired, and 60 (16%) were full-time employed. The duration of diagnosis in most of the participants was one to two years (n = 108, 28%), followed by six months to one year (n = 102, 27%), more than two years (n = 101, 26%), and less than six months (n = 73, 19%). Fatty liver disease (n = 108, 28%), hepatitis C (n = 83, 22%), alcoholic liver disease (n = 80, 21%), hepatitis B (n = 57, 15%), and autoimmune hepatitis (n = 56, 15%) were the most common liver conditions. Regarding treatment, 136 (35%) were receiving no treatment, 103 (27%) were managing with lifestyle or dietary modifications, 83 (22%) were committed to regular tests or check-ups, and 62 (16%) were on regular medication. Comorbidities were hypertension (n = 127, 33%), heart disease (n = 111, 29%), diabetes (n = 92, 24%), kidney issues (n = 36, 9%), and a low percentage reporting the lack of excess conditions (n = 18, 5%).

**Table 1:** Demographic characteristics of the participants (N = 384). N = number of participants; f = frequency; % = percentage. NAFLD: nonalcoholic fatty liver disease; NASH: nonalcoholic steatohepatitis.

Variable	f	%
Age	-	-
18-25 years	3	1
26-35 years	63	16
36-45 years	118	31
46-60 years	118	31
60 years and above	82	21
Gender	-	-
Male	170	44
Female	214	56
Marital status	-	-
Single	50	13
Married	109	28
Divorced	145	38
Widowed	80	21
Educational level	-	-
No formal education	27	7
Primary	67	17
Secondary	84	22
Higher secondary	88	23
Bachelor's degree	70	18
Master's degree	48	12
Employment status	-	-
Employed full-time	60	16
Self-employed	106	28
Unemployed	126	33
Retired	92	24
Duration since liver disease diagnosis	-	-
Less than 6 months	73	19
6 months-1 year	102	27
1-2 years	108	28
More than 2 years	101	26
Type of liver disease	-	-
Hepatitis B	57	15
Hepatitis C	83	22
Fatty liver disease (NAFLD/NASH)	108	28
Alcoholic liver disease	80	21
Autoimmune hepatitis	56	15
Current treatment status	-	-
Taking medication regularly	62	16
Undergoing regular lab tests/check-ups	83	22
On lifestyle/dietary changes only	103	27
Not on any treatment	136	35
Any comorbid condition?	-	-
Diabetes	92	24
Hypertension	127	33
Heart disease	111	29
Kidney problems	36	9
None	18	5

Table 2 presents the findings of normality testing of the MUIS and SEMCD-6 scales with two tests (Kolmogorov-Smirnov and Shapiro-Wilk tests). In the MUIS, the Kolmogorov-Smirnov statistic was 0.031, with a p-value of 0.200, and the Shapiro-Wilk statistic was 0.997, with a p-value of 0.623 ( df = 384 in both). Likewise, in the case of the SEMCD-6 (measuring confidence in managing chronic disease), the value of the Kolmogorov-Smirnov statistic was 0.035 (p-value = 0.200) and the Shapiro-Wilk statistic was 0.996 (p-value = 0.489) (df = 384). All the p-values are more than 0.05, and the assumption of normality holds on both scales, which means that parametric statistical tests are appropriate to conduct further tests.

**Table 2:** Tests of normality for the Mishel Uncertainty in Illness Scale and the Self-Efficacy for Managing Chronic Disease. df = degree of freedom; parametric test = p > 0.05; non-parametric test = p ≤ 0.05.

Variables	Kolmogorov-Smirnov			Shapiro-Wilk		
	Statistic	df	P	Statistic	df	p
Mishel Uncertainty in Illness Scale	0.031	384	0.200	0.997	384	0.623
Self-Efficacy for Managing Chronic Disease	0.035	384	0.200	0.996	384	0.489

Table 3 indicates that negative correlations existed between illness uncertainty and self-efficacy at three time points (T1, T2, and T3), which were statistically significant. The greater the levels of illness uncertainty, the lesser the self-efficacy (confidence in managing chronic disease) at time 1 (r = 0.288, p < 0.001), time 2 (r = -0.185, p < 0.001), and time 3 (r = -0.221, p < 0.001). Additionally, self-efficacy (confidence in managing chronic disease) scores were highly and significantly correlated across time, with the highest correlation observed between T1 and T3 (r = -0.634, p < 0.001). These results indicate that illness uncertainty predicts lower self-efficacy in managing chronic illness over time, durably, and substantially.

**Table 3:** Pearson's correlation coefficients between illness uncertainty and self-efficacy scores at T1, T2, and T3. ** P < 0.001 considered significant; correlation = Pearson’s.

Variable	1	2	3	4
Mishel Uncertainty in Illness Scale	-	-0.288^**^	-0.185^**^	-0.221^**^
Self-Efficacy for Managing Chronic Disease (Time 1)	-	-	-0.616^**^	-0.634^**^
Self-Efficacy for Managing Chronic Disease (Time 2)	-	-	-	-0.598^**^
Self-Efficacy for Managing Chronic Disease (Time 3)	-	-	-	-

Table 4 shows baseline gender-based comparisons between illness uncertainty and self-efficacy. The majority of the female participants had high levels of illness uncertainty (M = 73.45, SD = 6.45), compared to their male counterparts (M = 70.45, SD = 6.60) (t(382) = 4.37, p < 0.001), with a relatively large effect size (Cohen d = 0.46). In the same manner, a significant difference also existed in self-efficacy (confidence in managing chronic disease), where females (M = 36.25, SD = 7.20) scored higher than males (M = 33.10, SD = 7.55) (t(382) = -3.67, p < 0.001), with a moderate effect size (Cohen d = -0.43). These results suggest significant gender differences regarding the way people cope with the uncertainty of illness and confidence in their ability to take care of chronic conditions.

**Table 4:** Gender differences in illness uncertainty and self-efficacy at baseline. N = number of participants; M = mean; SD = standard deviation; LL = lower limit; UL = upper limit; CI = confidence interval; independent t-test; ** P < 0.001 considered significant.

Variable	Male (N = 170); M ± SD	Female (N = 214); M ± SD	t	p	95% CI, LL	UL	Cohen’s D
Mishel Uncertainty in Illness Scale	70.45 ± 6.60	73.45 ± 6.45	-4.37	<0.001^**^	-4.441	-1.542	-0.46
Self-Efficacy for Managing Chronic Disease (Time 1)	33.10 ± 7.55	36.25 ± 7.20	-3.67	<0.001^**^	-4.890	-1.413	-0.43

Table 5 presents the findings of a repeated-measures ANOVA analysis, which examines variations in self-efficacy over time on three occasions. When we analyzed it, it turned out that there was a significant main effect of the time variable upon self-efficacy (F (2) = 22.60, p < 0.001), with a small effect size (partial 2 = 0.019). Mean measures of self-efficacy (confidence in managing chronic disease) increased steadily across the time points, with values of 35.30 at baseline (T1), 36.18 at the midpoint (T2), and 36.49 at the endpoint (T3). These results indicate that the confidence (self-efficacy) of participants in managing chronic illness improved minimally yet noticeably during the study.

**Table 5:** Repeated measures ANOVA and descriptive statistics for self-efficacy across time (T1–T3). M = mean; SD = standard deviation; SS = sum of squares; MS = mean square; df = degrees of freedom; F = F-ratio; p = significance level. Each participant was assessed at three time points: T1, T2, and T3 (384 × 3 = 1152 observations). Self-efficacy was measured at three time points: baseline (T1), midpoint (T2), and endpoint (T3). A repeated measures ANOVA revealed a statistically significant main effect of time on self-efficacy (F = 22.60, p < 0.001, partial η² = 0.019). Greenhouse-Geisser correction was applied. The results were consistent across correction methods.

Variable	N	M ± SD	SS (time)	MS (time)	df	p	F	η2
Source	-	-	879.41	439.71	2	<0.001^**^	22.60	0.019
Time 1	1152	35.30 ± 7.561	-	-	-	-	-	-
Time 2	1152	36.18 ± 6.712	-	-	-	-	-	-
Time 3	1152	36.49 ± 7.013	-	-	-	-	-	-

Table 6 presents the scores of self-efficacy (confidence in managing chronic disease) at three time points (T1, T2, and T3) across the varying comorbid condition groups. According to the repeated measures ANOVA, there was a significant main effect of time (F (2, 2294) = 23.83, p < 0.001, η² = 0.020), which implies a general improvement in participants’ confidence (self-efficacy) over time. Additionally, a significant interaction was found between comorbid condition and time (F (8, 2294) = 3.77, p < 0.001, η² = 0.013), indicating that time differences in self-efficacy (confidence) became more pronounced when testing different health conditions. The most significant and steady gains were observed in participants with hypertension. In contrast, the baseline self-efficacy (confidence) was the lowest in those with kidney issues, and these gains were also substantial. These findings highlight the impact of comorbidity factors on the development of self-efficacy (confidence) in managing chronic illnesses.

**Table 6:** Descriptive statistics and repeated measures ANOVA for self-efficacy over time by comorbid conditions. N = number of participants; M = mean; SD = standard deviation; F = ratio of variance between groups to within groups; η2 = effect size. Means represent self-efficacy scores at each time point for each comorbid condition group. Repeated measures ANOVA was conducted using Greenhouse-Geisser correction. ** P < 0.001 considered significant.

Comorbid conditions	N	Time 1; M ± SD	Time 2; M ± SD	Time 3; M ± SD
Diabetes	276	33.62 ± 7.87	34.50 ± 6.56	34.18 ± 7.65
Hypertension	381	36.90 ± 7.49	37.60 ± 6.73	38.47 ± 6.77
Heart disease	333	36.34 ± 7.42	36.69 ± 6.86	36.78 ± 6.57
Kidney problems	108	31.42 ± 5.72	34.25 ± 5.60	34.92 ± 5.84
None	54	34.00 ± 5.94	35.56 ± 5.77	35.67 ± 5.87
Effect	F (df)	p	Partial η²	-
Time	23.83 (2, 2294)	<0.001^**^	0.020	-
Time × comorbid condition	3.77 (8, 2294)	<0.001^**^	0.013	-

Table 7 is a prospective linear regression equation, which forecasts self-efficacy (participants’ confidence in managing chronic disease) at time 3 (T3), depending on baseline (T1) resources. The model reveals high self-efficacy at T1 to be a significant predictor of self-efficacy (confidence) at T3 (B = 0.55, 0.62, p < 0.001), which shows high temporal stability of the measure. There was a significant negative relationship over time between illness uncertainty at T1 and self-efficacy (confidence) at T3 (B = -0.06, 3 = -0.18, p = 0.001). The nature of the liver disease was also positively associated, albeit with a minimal impact (B = 0.25, p = 0.038), and the presence of comorbidities was not a significant predictor (p = 0.153). Overall, baseline self-efficacy (confidence) and illness uncertainty were found to be the most significant predictors of future confidence (self-efficacy) in managing chronic illness.

**Table 7:** Prospective regression predicting self-efficacy at T3 from T1 illness uncertainty, T1 self-efficacy, liver disease type, and comorbidity. Constant = self-efficacy at time 3; B = coefficient; SE = standard error; β = standardized coefficient; LL = lower limit; UL = upper limit; CI = confidence interval; * p < 0.05; ** p < 0.01 considered significant.

Predictor	B	95% CI	-	SE	β	P
-	-	LL	UL	-	-	-
Constant	11.50	8.00	15.00	1.80	-	<0.001^**^
Illness uncertainty (t1)	–0.06	–0.10	–0.03	0.02	–0.18	0.001^**^
Self-efficacy (T1)	0.55	0.51	0.60	0.02	0.62	<0.001^**^
Type of liver disease	0.25	0.01	0.49	0.12	0.04	0.038^*^
Any comorbid condition?	0.20	–0.07	0.47	0.14	0.03	0.153

**Figure 1:**
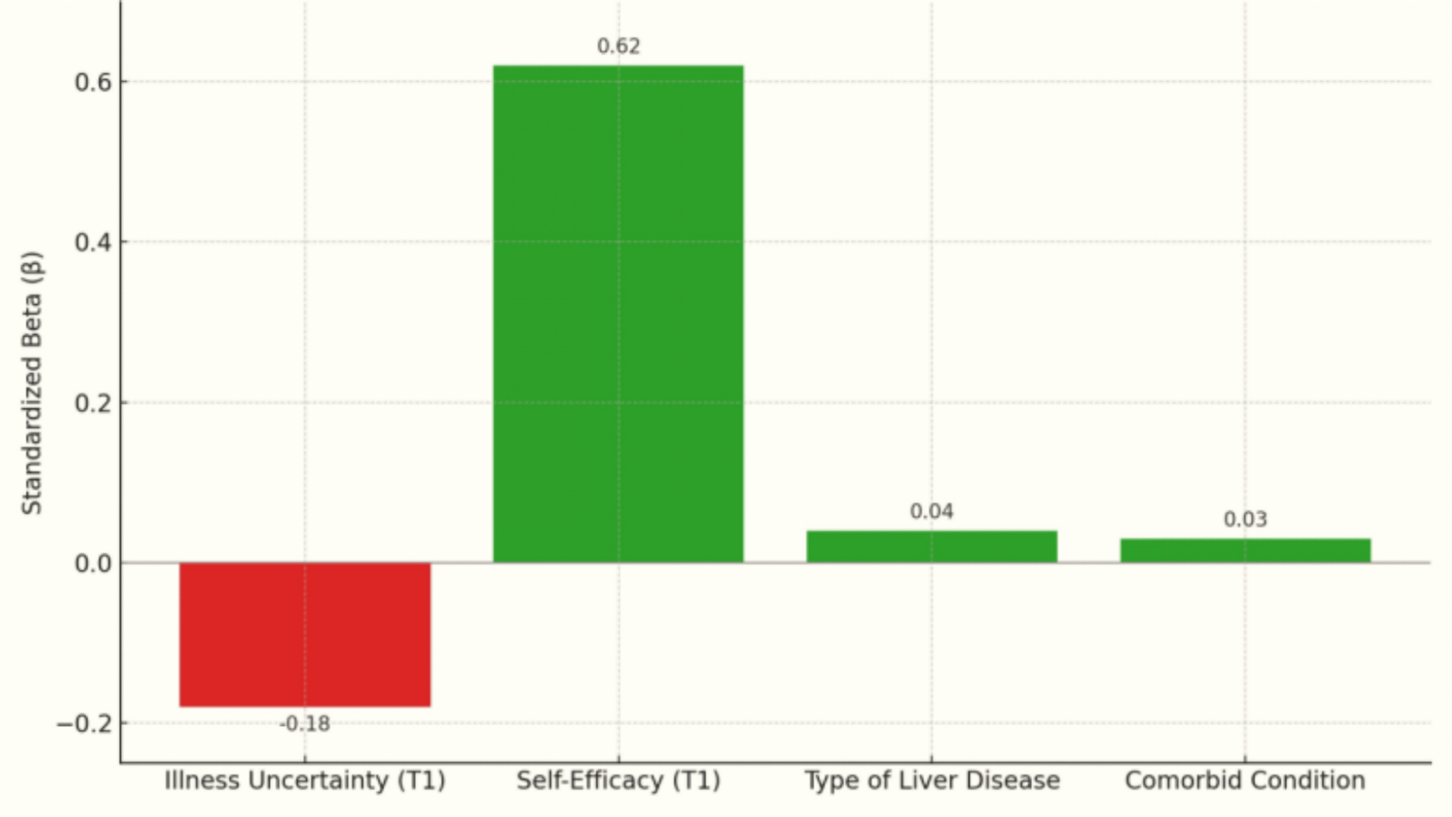
Standardized beta coefficients from a multiple regression predicting self-efficacy at time 3 from baseline illness uncertainty, self-efficacy, liver disease type, and comorbid conditions.

Figure 1 shows the results of a multiple regression model predicting self-efficacy (participants’ confidence in managing chronic disease) at time 3 (T3) based on baseline (T1) predictors: illness uncertainty, self-efficacy (confidence), type of liver disease, and comorbid conditions. Self-efficacy (confidence) at T1 (beta = 0.62) was the most influential predictor of future confidence (self-efficacy), showing a strong positive effect. The effect of illness uncertainty at T1 was moderate and negative (beta = -0.18), indicating that higher uncertainty at baseline is associated with lower confidence in managing chronic disease in the future. The predictive values of the type of liver disease (B = 0.04) and comorbid conditions (B = 0.03) were small and positive. Overall, baseline self-efficacy (confidence) and illness uncertainty were the most significant predictors of long-term confidence in managing chronic illness in this clinical population.

Table 8 presents the distribution of various comorbid conditions and types of liver diseases across different age groups. Chi-square tests revealed statistically significant correlations between age and comorbid conditions (χ² = 35.4, p = 0.003) and between age and liver disease types (χ² = 35.8, p = 0.003). Adults (36-60+ years) were more likely to give multiple comorbidities during reporting, especially diabetes, hypertension, and heart disease. Likewise, fatty liver disease, hepatitis C, and alcoholic liver disease were familiar to people above 36 years. These data indicate that increasing age is considerably linked with the augmented weight of comorbidity and CLDs among the research population.

**Table 8:** Descriptive statistics of demographic variables (age, comorbid conditions, and type of liver disease). f = frequency; % = percentage; df = degree of freedom; x2 = effect size; p = level of significance. P-values calculated using the chi-square test. The significance level is set at p < 0.05. Comorbid conditions are not mutually exclusive. Participants may have reported more than one condition. NAFLD: nonalcoholic fatty liver disease; NASH: nonalcoholic steatohepatitis.

Variables	f	Diabetes	Comorbid conditions: hypertension	Heart disease	Kidney problems	None	df	p	x^2^	Hepatitis B	Hepatitis C	Type of liver disease: fatty liver disease (NAFLD/NASH)	Alcoholic liver disease	Autoimmune hepatitis	df	p	x^2^
Age	-	-	-	-	-	-	16	0.003^**^	35.4	-	-	-			16	0.003^**^	35.8
18-25 years	3	0	1	1	0	1	-	-	-	2	1	0	0	0	-	-	-
26-35 years	63	24	13	21	3	2	-	-	-	15	11	14	11	12	-	-	-
36-45 years	118	18	47	39	11	3	-	-	-	15	32	33	28	10	-	-	-
46-60 years	118	28	38	31	10	11	-	-	-	9	20	46	26	17	-	-	-
60 years and above	82	22	28	19	12	1	-	-	-	16	19	15	15	17	-	-	-

## Discussion

This study aimed to examine the prospective association between illness uncertainty and self-management behaviors in patients with early-stage CLD; however, the follow-up period was limited to six months, which may not fully capture longer-term changes in self-efficacy and self-management behaviors. Our result showed that higher illness uncertainty is substantially related to low self-efficacy at all time points. This observation aligns with past literature, in which illness uncertainty was negatively associated with self-efficacy, which in turn positively predicted their sense of shared decision-making [15].

Our study revealed higher levels of illness uncertainty among female subjects compared to males. This result is similar to other chronic illness populations, where females also had greater illness uncertainty, thus demonstrating how different genders may have varying coping behavior with chronic illnesses [16]. However, these gender differences were observed without adjusting for potential confounders, such as education, employment status, or social support, which may have influenced the results. This further reaffirms the necessity of examining gender issues that play a role in illness perception and management. In our research, the self-efficacy of female participants was found to be significantly higher than that of male participants. This, however, is contrary to other studies, which consistently show that males tend to score higher in self-efficacy than their female counterparts in many areas [17]. Such disparity could be caused by differences in the type of illness, settings of care, or gender-specific roles that affect confidence in self-management.

Our results showed that self-efficacy improved significantly over time, suggesting that a patient's confidence in managing the illness may also increase over time. This aligns with earlier evidence among chronic illness populations, where self-efficacy is positively correlated with modifiable factors, including physical activity, social support, and a beneficial view of the illness [18].

In our analysis, self-efficacy increased with time in various comorbid groups, especially among participants diagnosed with kidney-related conditions. This result aligns with past studies, which have shown that a greater level of knowledge about the disease is linked to stronger self-efficacy and quality of life in patients with hypertensive nephropathy [19], highlighting the fact that self-efficacy has a modifiable component in chronic painful diseases.

In our research, baseline self-efficacy proved to be the most influential determinant of future self-efficacy, aligning with existing research that has shown a correlation between greater daily self-efficacy and improved long-term health practices, such as physical activity [20]. The results support the notion that early self-efficacy is crucial in determining long-term self-management outcomes. In our analysis, baseline illness uncertainty was the strongest predictor of poorer subsequent self-efficacy, whereas baseline self-efficacy was the strongest predictor of good subsequent self-efficacy. Likewise, in a prior study, it was found that higher levels of illness uncertainty corresponded to decreased self-efficacy [15], suggesting that minimizing uncertainty is necessary to enhance confidence in patients. The type of liver disease had a more minor but significant positive longitudinal relationship to self-efficacy in our research. This observation aligns with the findings of previous studies, which have shown that an increase in self-efficacy is associated with an increase in perceived illness knowledge and treatment efficacy. Consequently, disease-specific characteristics and perceptions may also impact self-management [21]. Comorbidity did not significantly contribute to future self-efficacy in our study; therefore, it did not serve as a significant predictor of self-efficacy levels in patients with various health conditions. This supports previous findings that self-efficacy has been identified as an important predictor of health behavior in numerous situations, leading to its overrepresentation in comorbidities [22].

We found that older participants were more likely to experience hypertension and other comorbidities as the cumulative burden of disease increases with age. This result is consistent with prior studies, which emphasized that hypertension has become a prevalent comorbid condition in ageing populations, with other decades-long conditions frequently co-occurring in people with diabetes and cardiovascular disease [23]. Within our study, the tendency to have fatty liver disease was significantly higher among the participants of older age, as has been previously shown in the literature regarding the incidence of NAFLD, which was 40% in participants aged 60 years and above. This confirms the relationship between the rise in age and the risk of fatty liver disease [24].

Limitations

Despite its strong points, this research study has some limitations. To begin with, the use of a convenience sampling technique can limit the applicability of the results to the broader population of patients with early-stage CLD. Second, a notably high proportion of participants were divorced (38%), which is unusual compared to the general population and may have influenced psychological factors and self-efficacy, further limiting representativeness. Third, the assessments were presented using self-reported questionnaires, which can be biased by social desirability effects or memory errors. Fourth, the study only tracked participants for six months as the follow-up period. In contrast, a longer follow-up would be needed to observe more substantial changes in self-management behavior and health outcomes. Moreover, the study was conducted at a single center, further limiting the generalizability of the findings. Additionally, potential residual confounding variables, including unmeasured factors related to healthcare access or psychosocial support, could have influenced the observed associations. Finally, the research did not evaluate external aspects such as structured psychological interventions, which could mediate the impact of illness uncertainty on self-efficacy.

Future directions

Future studies should investigate specific interventions to reduce the uncertainty of illness, such as psychoeducational courses, counseling, or directed patient education, to identify their direct effects on enhancing self-management behavior in early-stage CLD individuals. Prolonged longitudinal studies (12 months or longer) and varied clinical environments may help reveal more about the temporal dynamics of uncertainty and its impact on the disease course. Additional psychosocial variables, including depression, health literacy, and social support, should also be examined to better understand factors influencing self-management behaviors.

## Conclusions

This study demonstrates that higher illness uncertainty is associated with lower self-efficacy in patients with early-stage CLD. Although self-efficacy improved slightly over the six-month follow-up, participants with greater baseline uncertainty remained less confident in managing their condition. Interventions aimed at reducing illness uncertainty may help enhance self-efficacy, potentially supporting better self-management behaviors. Incorporating psychological assessment and counseling into routine hepatology care may facilitate patient-centered support during the early stages of disease. Observed associations should be interpreted cautiously, as causal inferences cannot be drawn from this observational study.

## Appendices

**Table 9:** Demographic information questionnaire.

Items	Responses
Age	-
Gender	-
Marital status	-
Educational level	-
Employment status	-
Duration since liver disease diagnosis	-
Type of liver disease (as reported by physician)	-
Current treatment status	-
Any co-morbid condition? (Choose all that apply)	

**Table 10:** Mishel Uncertainty in Illness Scale – Community Form (MUIS-C). Reproduced from Mishel MH (1981) [13]. Permission for use was obtained from the copyright holder, the University of North Carolina at Chapel Hill. The instrument is presented in its original, unmodified form and is authorized for use in academic research purposes.

Items	Responses
I don’t know what is wrong with me.	-
I have a lot of questions without answers.	-
I am unsure if my illness is getting better or worse.	-
It is unclear to me how bad my pain will be.	-
The explanations they give about my condition seem hazy to me.	-
The purpose of each treatment is clear to me.	-
My symptoms continue to change unpredictably.	-
I understand everything explained to me.	-
The doctors say things to me that have many meanings.	-
My treatment is too complex to figure out.	-
It is difficult to know if the treatments or medications I am getting are helping me.	-
Because of the unpredictability of my illness, I cannot plan for the future.	-
The course of my illness keeps changing. I have good days and bad days.	-
I have been given differing opinions about what is wrong with me.	-
It is not clear what is going to happen to me.	
The results of my tests are inconsistent.	
The effectiveness of the treatment is undermined.	
Because of the treatment, what I can do and cannot do keeps changing.	
I’m certain they will not find anything else wrong with me.	
The treatment I am receiving has a known probability of success.	
They have not given me a specific diagnosis.	
The seriousness of my illness has been determined.	
The doctors and nurses use everyday language so I can understand what they are saying.	

**Table 11:** Self-Efficacy for Managing Chronic Disease (SEMCD-6). Data adapted from the scale developed by Kate Lorig and colleagues (2001) [14], which is publicly available.

Items	Responses
How confident do you feel that you can keep the fatigue caused by your disease from interfering with the things you want to do?	-
How confident do you feel that you can keep the physical discomfort or pain of your disease from interfering with the things you want to do?	-
How confident do you feel that you can keep the emotional distress caused by your disease from interfering with the things you want to do?	-
How confident do you feel that you can keep any other symptoms or health problems you have from interfering with the things you want to do?	-
How confident do you feel that you can do the different tasks and activities needed to manage your health condition so as to reduce your need to see a doctor?	-
How confident do you feel that you can do things other than just taking medication to reduce how much your illness affects your everyday life?	-
